# Coexisting schwannoma of the gallbladder and sarcoidosis: a case report

**DOI:** 10.1186/s40792-020-00839-4

**Published:** 2020-04-19

**Authors:** Takuya Tajiri, Hiromitsu Hayashi, Takaaki Higashi, Takanobu Yamao, Toru Takematsu, Norio Uemura, Kensuke Yamamura, Katsunori Imai, Yo-ichi Yamashita, Hideo Baba

**Affiliations:** Department of Gastroenterological Surgery, Kumamoto University, 1-1-1 Honjo, Kumamoto, 860-8556 Japan

**Keywords:** Schwannoma, Gallbladder, Sarcoidosis, Steroid, Case report

## Abstract

**Background:**

Schwannomas originate from Schwann cells, which are constituents of peripheral nerve sheaths, and can occur anywhere in the body at any age. Most occur in soft tissues such as subcutaneous tissues and muscles, occurrence in the abdominal cavity being relatively rare. In particular, schwannomas of the gallbladder are extremely rare. We herein report a rare case of a schwannoma that coexisted with systemic sarcoidosis and presented as a steroid-resistant mass in the gallbladder wall.

**Case presentation:**

A 40-year-old woman was found to have thickening of the gallbladder wall during a routine medical examination and was referred to our hospital, where she was found to have granular shadows in the lungs; mediastinal, cervical, intraperitoneal, and inguinal lymphadenopathy; parotid gland enlargement; and an erythematous skin rash. She was diagnosed as having systemic sarcoidosis by transbronchial lung biopsy and bronchoalveolar lavage. All her systemic mass lesions except for the one in the gallbladder resolved or became smaller with steroid treatment. The steroid-resistant gallbladder lesion showed enhancement on contrast-enhanced computed tomography and was shown by endoscopic ultrasonography to be a 30-mm-diameter gallbladder wall lesion. We performed laparoscopic cholecystectomy, which resulted in diagnosis of the steroid-resistant tumor as a schwannoma. Five months after surgery, the patient’s prednisolone dosage had been gradually reduced to 5 mg/day and she was doing well with no evidence of recurrence.

**Conclusion:**

Resection of a steroid-resistant tumor resulted in diagnosis of schwannoma, enabling reduction in the patient’s steroid dosage for sarcoidosis.

## Background

Schwannomas originate from Schwann cells, which are constituents of peripheral nerve sheaths, and can occur anywhere in the body at any age [1]. Most occur in soft tissues such as subcutaneous tissues and muscles, occurrence in the abdominal cavity being relatively rare. In particular, schwannomas of the gallbladder are extremely rare. We herein report a rare patient with a schwannoma of the gallbladder that coexisted with systemic sarcoidosis and presented as a steroid-resistant mass in the gallbladder wall.

## Case presentation

**Fig. 1:**
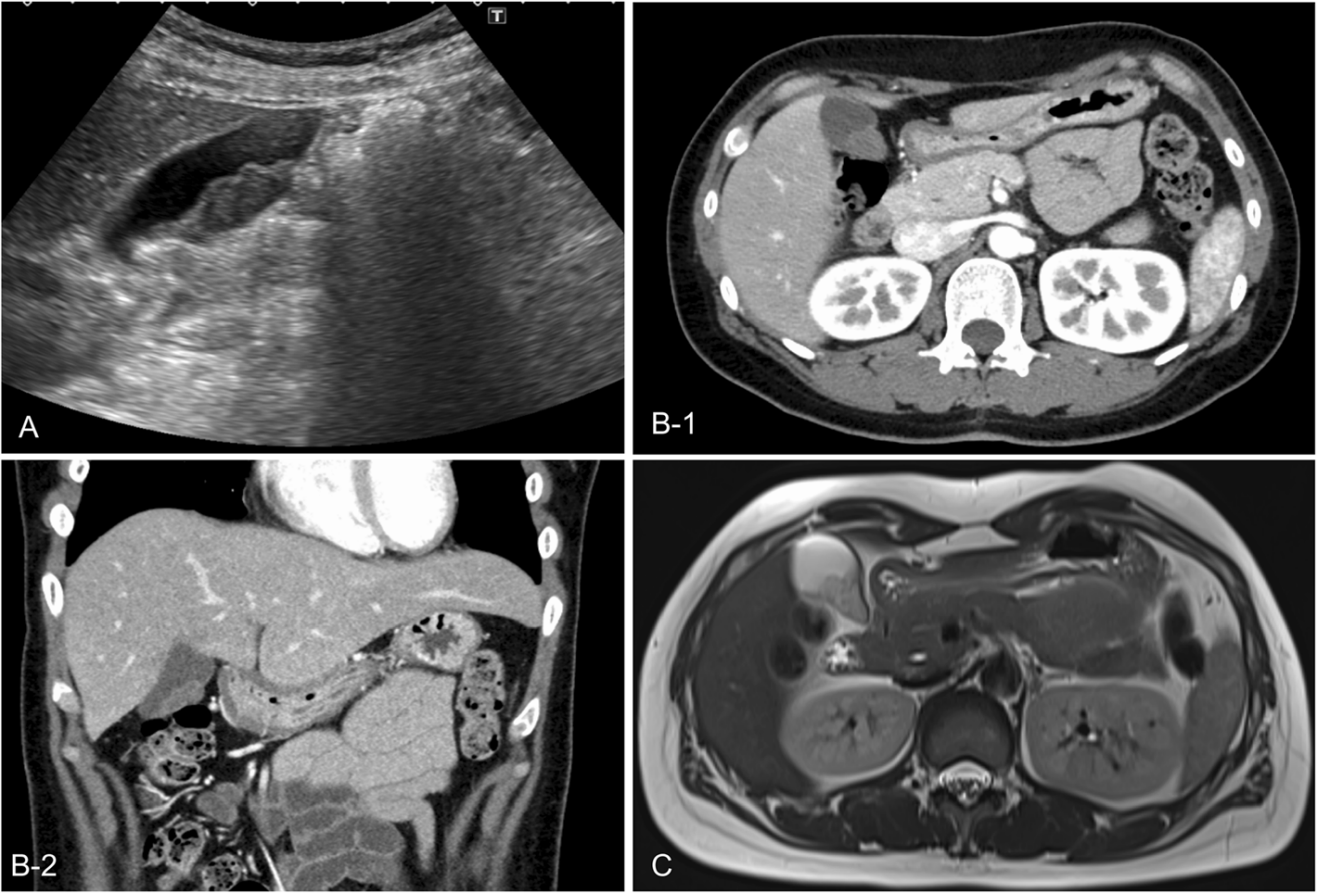
**a** US image showing a mass in the gallbladder. **b-1** CECT scan image showing a mass in the gallbladder wall that takes up contrast. **b-2** CECT scan image showing a mass in the gallbladder wall that takes up contrast. **c** MRI T2 image showing a gallbladder lesion with hypointensity

**Fig. 2:**
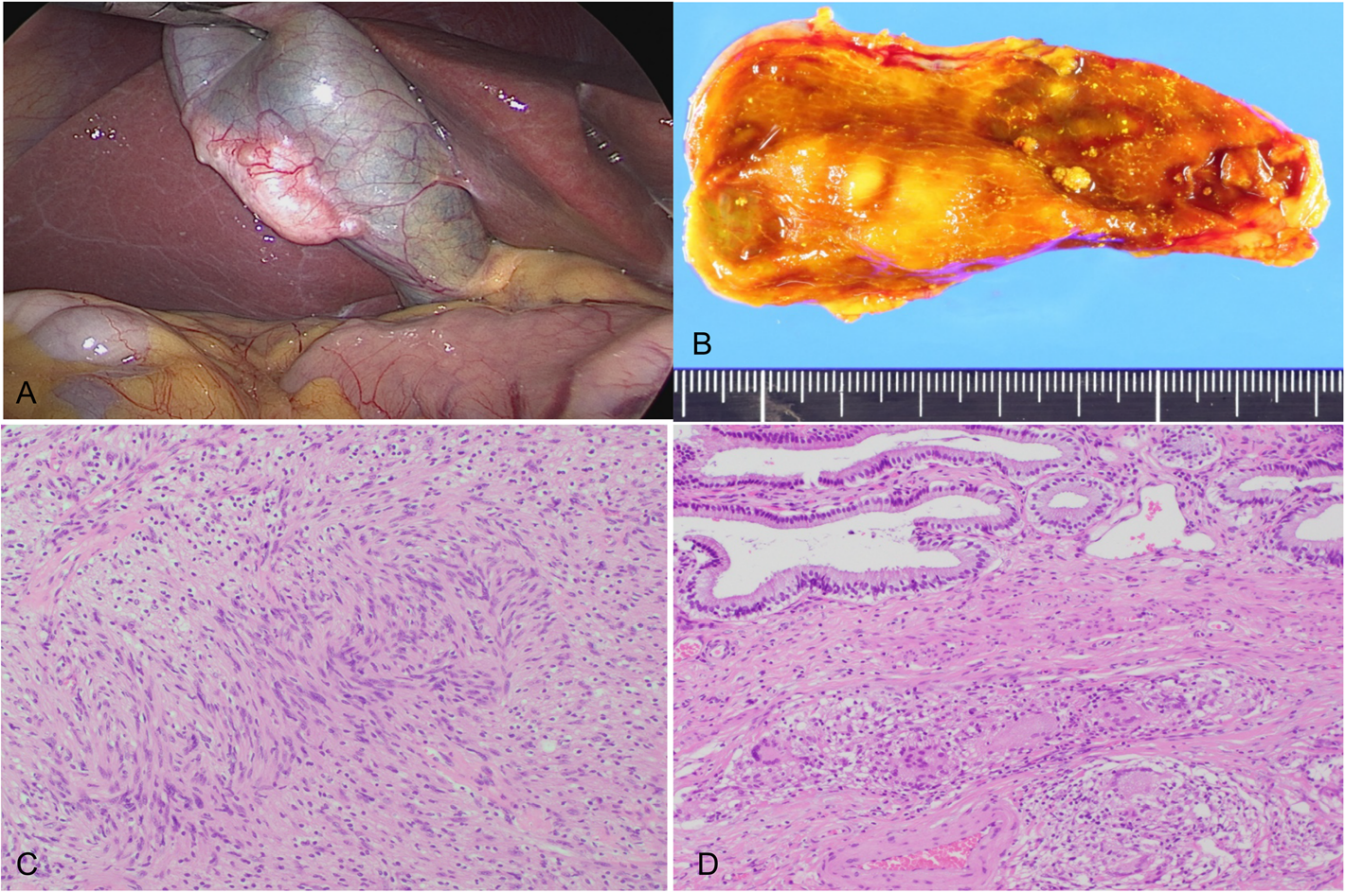
**a** Photograph of gallbladder during laparoscopy. **b** Photograph of the resected specimen showing an elastic hard mass under the gallbladder serosa. **c** Photomicrograph of a section of the schwannoma (hematoxylin-eosin [HE] stain, × 100). **d** Photomicrograph of HE-stained section of the tumor showing non-necrotic granulation consistent with sarcoidosis (× 100)

An asymptomatic 40-year-old woman was found to have gallbladder wall thickening during a routine medical examination. There was no medical history except for uveitis with recent blurred vision. She was taking no medications and had no history of allergy. Her vital signs were unremarkable. On physical examination, cervical and inguinal lymphadenopathy, parotid gland enlargement, and an erythematous skin rash were detected. Soluble interleukin-2 receptor and angiotensin-converting enzyme concentrations were 2431 U/mL (normal range 121–613) and 27.6 U/L (normal range 8.3–21.4), respectively. Other laboratory data, including tumor markers such as carcinoembryonic antigen (CEA) and carbohydrate antigen 19-9 (CA19-9), were within the normal range. Abdominal ultrasonography revealed a hypoechoic mass (30-mm diameter) with no posterior echo enhancement on the abdominal side of the body of the gallbladder (Fig. 1a). The hypoechoic mass had slightly irregular borders, was elliptical in shape, and was almost uniform in consistency. In addition, another hypoechoic mass (20-mm diameter) with a clear circular border was noted in the hepatoduodenal ligament and suspected of being an enlarged lymph node. Contrast-enhanced computed tomography (CECT) revealed a mass lesion of the gallbladder wall with a contrast effect (Fig. 1b). Also, bilateral parotid gland enlargement and enlargement of multiple lymph nodes around the right supraclavicular, mediastinum, lower thoracic para-esophageal, common hepatic artery, and hepatoduodenal ligament were detectable. These lymph nodes showed uptake on positron emission tomography-computed tomography (PET-CT); however, the gallbladder lesion did not. This lesion revealed hypointensity on T1 and T2 images and no diffusion limitation on magnetic resonance imaging (MRI) (Fig. 1c). Transbronchial lung biopsy (TBLB) and bronchoalveolar lavage (BAL) were performed. Finally, our patient was diagnosed as having systemic sarcoidosis by biopsy. She was prescribed prednisolone (PSL) 25 mg/day for the systemic sarcoidosis, resulting in complete resolution or reduction in size of all mass lesions except for the gallbladder lesion. Endoscopic ultrasonography (EUS) of the gallbladder lesion showed that the gallbladder lumen was intact and did not identify any mucosal lesions. It was concluded that she had a steroid-resistant lymph node in the gallbladder wall. Excision of the gallbladder lesion by laparoscopic cholecystectomy was therefore performed (Fig. 2a). The patient was discharged on postoperative day 5 with no postoperative complications. The resected specimen measured 37 × 17 mm, was yellowish-white in color, and contained an elastic hard mass under the gallbladder serosa (Fig. 2ba). Histopathological examination showed that the tumor was composed of spindle cells in an irregular palisade arrangement and Verocay body formation with no evidence of malignancy (Fig. 2c). The spindle cells were positive for S-100 protein on immunostaining. In addition, non-necrotic granulation, which is consistent with sarcoidosis, was found in part of the lamina propria (Fig. 2d). Finally, the gallbladder lesion was diagnosed as a schwannoma. Five months after surgery, the PSL dosage had been gradually reduced to 5 mg/day and the patient was doing well with no evidence of recurrence.

## Discussion

We here describe a patient with sarcoidosis and a coexisting schwannoma that presented as a steroid-resistant mass in the gallbladder wall. Intra-abdominal schwannomas have reportedly occurred in the intrahepatic duodenal ligament [2], pancreas [3], small mesentery [4], and colon [5]. To the best of our knowledge, only ten schwannomas in the gallbladder, including the present case, have been reported [6–9]. Schwannomas are histopathologically composed of spindle cells in a nuclear palisade arrangement and Verocay body formation [10]. On immunostaining, the spindle cells are characteristically strongly positive for S-100 protein and negative for desmin, smooth muscle myosin, SMA, c-KIT, CD34, and CD117 [11]. As for clinical features, schwannomas that develop in the abdominal cavity are often asymptomatic for a long time, eventually presenting with symptoms of compression of surrounding organs [2].

Schwannomas arising in the digestive tract can be easily accessible to endoscopic biopsy, whereas it is difficult to obtain a biopsy of the gallbladder to make a definitive diagnosis [2]. Schwannomas have no distinctive imaging findings and the preoperative diagnosis is often difficult. Multiple imaging modalities such as contrast-enhanced US, CT, and MRI may be useful in making a diagnosis. Color Doppler ultrasound reportedly shows well-defined hypodense lesions with no echoic enhancement [12]. CECT shows well-defined hypodense lesions with encapsulation and/or cystic degeneration [11], and MRI shows hypointensity on T1-weighted images and lack of homogeneity and hyperintensity on T2-weighted images [13, 14]. Contrast-enhanced US and CECT can evaluate the intra-tumor blood flow, enabling differentiation from malignant tumors [6].

In contrast, sarcoidosis is an inflammatory disease that is characterized by formation of granulomas (small nodules of immune cells) in the lungs, lymph nodes, and other organs. Sarcoidosis may be acute and resolve spontaneously, or be chronic and progressive [15]. More than 80% of cases occur in adults between 20 and 50 years of age [16]. Treatment is not indicated for patients with asymptomatic sarcoidosis because spontaneous resolution is common [16]. Treatment with corticosteroids should be considered for patients with significant symptomatic or progressive pulmonary disease or serious extrapulmonary disease [17]. An international consensus statement recommended prednisone (or its equivalent) at a starting dosage of 20 to 40 mg per day for 4 to 6 weeks [16]. If the patient’s condition is stable or improved, the dosage should be tapered slowly to approximately 5 to 10 mg per day.

To the best of our knowledge, no relationship between sarcoidosis and schwannoma has been documented. The differential diagnosis for both sarcoidosis and schwannoma is broad because of the nonspecific symptoms and diverse clinical presentations. Because many other diseases present with similar clinical, radiologic, and pathologic findings, infections and malignancy (e.g., lymphoma) should be ruled out if suspected [17].

Our patient’s schwannoma was complicated by her coexisting systemic sarcoidosis, which is an extremely rare combination. Because all lesions except for the one in the gallbladder were responsive to steroids, it was resected to obtain an accurate diagnosis, the rationale being that if it was not a sarcoid lesion, we could reduce her dosage of steroids.

In addition, in this case, the excised specimen showed a mixture of spindle-shaped lesions indicating a schwannoma and non-necrotic granulomatous lesions indicating sarcoidosis, which may have a component of neurosarcoidosis as reported by Bangiyev et al. [18].

## Conclusions

Schwannomas in the abdominal cavity, including in the gallbladder, are rare. We excised our patient’s steroid-resistant gallbladder mass to obtain a definitive diagnosis because she also had steroid-responsive systemic sarcoidosis. Excision provided an accurate diagnosis, resulting in more rational treatment.

## Data Availability

All data generated or analyzed during this study are included in this published article.

## Abbreviations

CECT: Contrast-enhanced computed tomography
PET-CT: Positron emission tomography-computed tomography
MRI: Magnetic resonance imaging
TBLB: Transbronchial lung biopsy
BAL: Bronchoalveolar lavage
PSL: Prednisolone
EUS: Endoscopic ultrasonography
US: Ultrasonography
